# 2-Amino-4-(4-fluoro­phen­yl)-6-meth­oxy-4*H*-benzo[*h*]chromene-3-carbonitrile

**DOI:** 10.1107/S1600536812023021

**Published:** 2012-05-31

**Authors:** Al-Anood M. Al-Dies, Abdel-Galil E. Amr, Ahmed M. El-Agrody, Tze Shyang Chia, Hoong-Kun Fun

**Affiliations:** a Chemistry Department, Faculty of Science, King Khalid University, 9004 Abha, Saudi Arabia; b Drug Exploration & Development Chair (DEDC), College of Pharmacy, King Saud University, Riyadh 11451, Saudi Arabia; c Applied Organic Chemistry Department, National Research Center, Dokki 12622, Cairo, Egypt; d X-ray Crystallography Unit, School of Physics, Universiti Sains Malaysia, 11800 USM, Penang, Malaysia

## Abstract

In the title mol­ecule, C_21_H_15_FN_2_O_2_, the dihedral angle between the fluoro-substituted benzene ring and the mean plane of the 4*H*-benzo[*h*]chromene ring system [maximum deviation = 0.109 (2) Å] is 83.35 (7)°. The pyran ring adopts a slight sofa conformation with the tertiary C(H) atom forming the flap. The meth­oxy group is slightly twisted from the attached benzene ring of the 4*H*-benzo[*h*]chromene moiety [C—O—C—C = −4.3 (3)°]. In the crystal, mol­ecules are linked by inter­molecular N—H⋯N hydrogen bonds into infinite wave-like chains along the *b* axis. The crystal packing is further stabilized by π–π inter­actions [centroid–centroid distance = 3.7713 (9) Å].

## Related literature

For the synthesis of 4*H*-chromene derivatives, see: Sayed *et al.* (2000 ▶); Bedair *et al.* (2001 ▶); El-Agrody *et al.* (2000 ▶, 2002 ▶). For the chemical and pharmacological properties of 4*H*-chromene and fused 4*H*-chromene derivatives, see: El-Agrody *et al.* (2000 ▶); Abd-El-Aziz *et al.* (2004 ▶, 2007 ▶); Sabry *et al.* (2011 ▶). For ring puckering parameters, see: Cremer & Pople (1975 ▶).
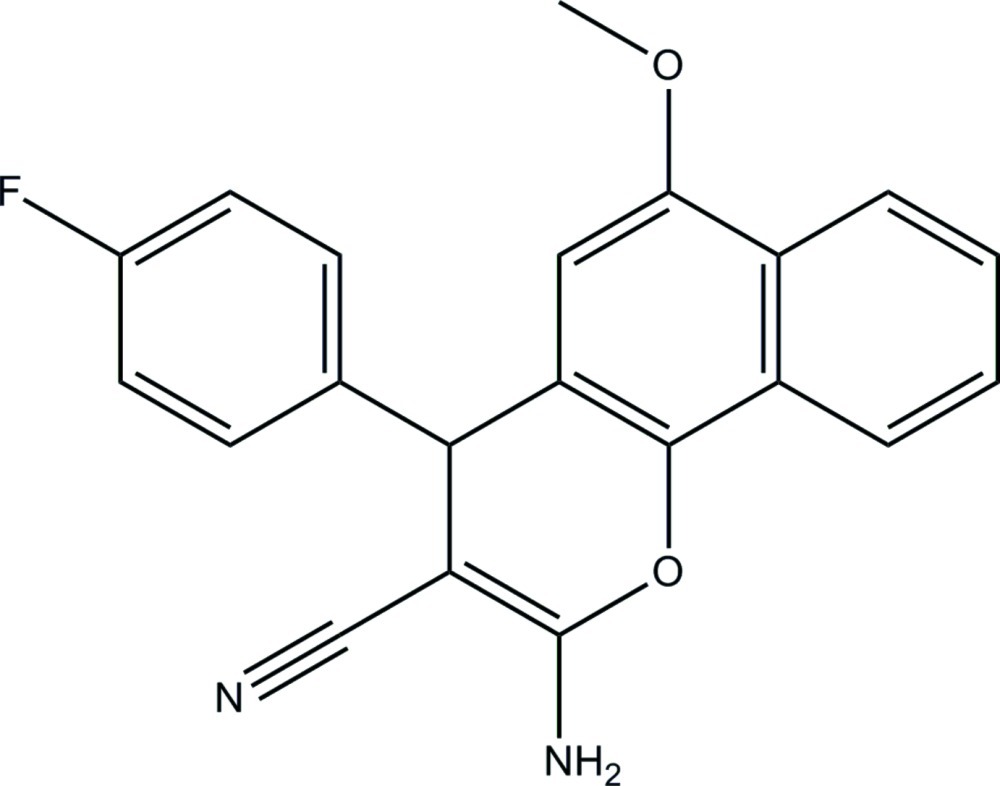


## Experimental

### 

#### Crystal data

C_21_H_15_FN_2_O_2_
*M* *_r_* = 346.35
Monoclinic, 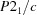

*a* = 12.6336 (4) Å
*b* = 11.9333 (3) Å
*c* = 12.0471 (4) Å
β = 113.581 (2)°
*V* = 1664.56 (9) Å^3^
*Z* = 4
Cu *K*α radiation
μ = 0.81 mm^−1^
*T* = 296 K
0.88 × 0.68 × 0.06 mm

#### Data collection

Bruker SMART APEXII CCD area-detector diffractometer
Absorption correction: multi-scan (*SADABS*; Bruker, 2009 ▶) *T*_min_ = 0.537, *T*_max_ = 0.953
11390 measured reflections
3199 independent reflections
2770 reflections with *I* > 2σ(*I*)
*R*_int_ = 0.035

#### Refinement

*R*[*F* ^2^ > 2σ(*F* ^2^)] = 0.047
*wR*(*F* ^2^) = 0.132
*S* = 1.05
3199 reflections
245 parameters
H atoms treated by a mixture of independent and constrained refinement
Δρ_max_ = 0.22 e Å^−3^
Δρ_min_ = −0.21 e Å^−3^

### 

Data collection: *APEX2* (Bruker, 2009 ▶); cell refinement: *SAINT* (Bruker, 2009 ▶); data reduction: *SAINT*; program(s) used to solve structure: *SHELXTL* (Sheldrick, 2008 ▶); program(s) used to refine structure: *SHELXTL*; molecular graphics: *SHELXTL*; software used to prepare material for publication: *SHELXTL* and *PLATON* (Spek, 2009 ▶).

## Supplementary Material

Crystal structure: contains datablock(s) global, I. DOI: 10.1107/S1600536812023021/lh5476sup1.cif

Structure factors: contains datablock(s) I. DOI: 10.1107/S1600536812023021/lh5476Isup2.hkl

Supplementary material file. DOI: 10.1107/S1600536812023021/lh5476Isup3.cml

Additional supplementary materials:  crystallographic information; 3D view; checkCIF report

## Figures and Tables

**Table 1:** Hydrogen-bond geometry (Å, °)

*D*—H⋯*A*	*D*—H	H⋯*A*	*D*⋯*A*	*D*—H⋯*A*
N1—H2*N*1⋯N2^i^	0.89 (2)	2.17 (2)	3.054 (2)	175 (2)

Symmetry code: (i) 
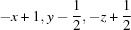
.

**Fig. 1.**
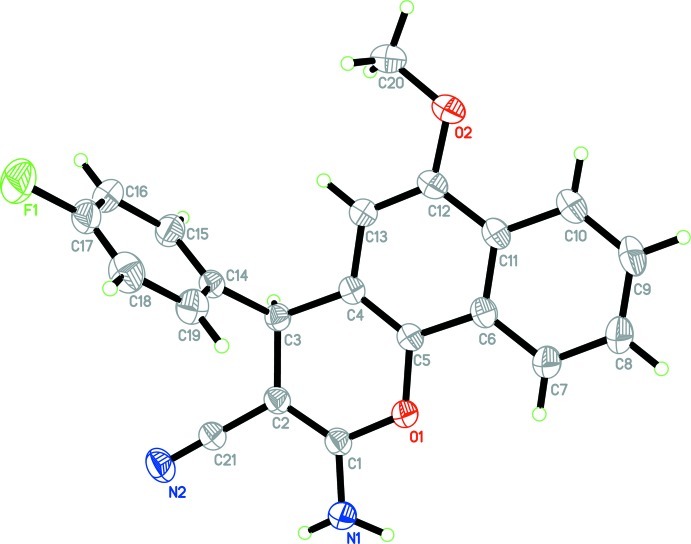
The molecular structure of the title compound with 30% probability displacement ellipsoids.

**Fig. 2.**
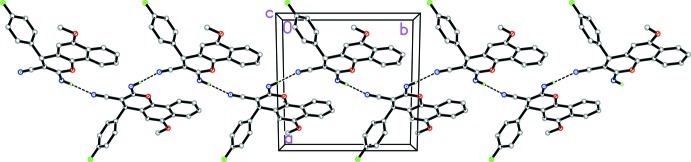
The crystal packing of the title compound. The dashed lines represent the hydrogen bonds. For clarity, hydrogen atoms not involved in hydrogen bonding have been omitted.

## supplementary crystallographic information

### Comment

In our previous work, we have reported the synthesis of 4*H*-chromene derivatives using α-cyanocinnamonitriles and ethyl α-cyanocinnamates (Sayed *et al.*, 2000; Bedair *et al.*, 2001; El-Agrody *et al.*, 2000, 2002), study of their characterization and evaluation of their antimicrobial and antitumor activities. In continuation of our interest in the chemical and pharmacological properties of 4*H*-chromene and fused 4*H*-chromene derivatives (El-Agrody *et al.*, 2000; Abd-El-Aziz *et al.*, 2004, 2007; Sabry *et al.*, 2011), we report herein the crystal structure of the title compound.

The asymmetric unit of the title compound is shown in Fig. 1. The fluoro-substituted benzene ring (C14–C19) is approximately perpendicular to the 4*H*-benzo[*h*]chromene ring system [O1/C1–C13, maximum deviation = 0.109 (2) Å at atom C3] as indicated by the dihedral angle of 83.35 (7)°. The pyran ring (O1/C1–C5) adopts slight sofa conformation [puckering parameters (Cremer & Pople, 1975), *Q* = 0.0980 (16) Å, θ = 69.5 (9)° and φ = 167.7 (10)°] with C3 as the flap atom. The methoxy group (C20/O2) is slightly twisted from the attached benzene ring (C4–C6/C11–C13) of the 4*H*-benzo[*h*]chromene moiety with torsion angle C20—O2—C12—C13 of -4.3 (3)°.

In the crystal (Fig. 2), molecules are linked by intermolecular N1—H2N1···N2^i^ hydrogen bonds (Table 1) into infinite wave-like chains along *b* axis. The crystal packing is further stabilized by π–π interaction with *Cg*1-*Cg*1 distance of 3.7713 (9) Å, where *Cg*1 is the centroid of O1/C1–C5 ring [symmetry code: 1-*x*, 1-*y*, -*z*].

### Experimental

A solution of 4-methoxy-1-naphthol (0.01 mol) in EtOH (30 ml) was treated with α-cyano-*p*-fluorocinnamonitrile (0.01 mol) and piperidine (0.5 ml). The reaction mixture was heated until complete precipitation occurred (reaction time: 60 min). The solid product formed was collected by filtration and recrystallized from ethanol to give the title compound. *M.p.*: 493–494 K.

### Refinement

The atoms H2N1 and H1N1 were located in a difference fourier map and refined freely [N—H = 0.89 (2) and 0.90 (2) Å]. The remaining H atoms were positioned geometrically [C—H = 0.93, 0.96 and 0.98 Å] and refined using a riding model with *U*_iso_(H) = 1.2 or 1.5 *U*_eq_(C). A rotating group model was applied to the methyl group.

### Crystal data

**Table:**

C_21_H_15_FN_2_O_2_	*F*(000) = 720
*M**_r_* = 346.35	*D*_x_ = 1.382 Mg m^−^^3^
Monoclinic, *P*2_1_/*c*	Cu *K*α radiation, λ = 1.54178 Å
Hall symbol: -P 2ybc	Cell parameters from 3014 reflections
*a* = 12.6336 (4) Å	θ = 3.8–71.5°
*b* = 11.9333 (3) Å	µ = 0.81 mm^−^^1^
*c* = 12.0471 (4) Å	*T* = 296 K
β = 113.581 (2)°	Plate, yellow
*V* = 1664.56 (9) Å^3^	0.88 × 0.68 × 0.06 mm
*Z* = 4	

### Data collection

**Table:**

Bruker SMART APEXII CCD area-detector diffractometer	3199 independent reflections
Radiation source: fine-focus sealed tube	2770 reflections with *I* > 2σ(*I*)
Graphite monochromator	*R*_int_ = 0.035
φ and ω scans	θ_max_ = 71.9°, θ_min_ = 3.8°
Absorption correction: multi-scan (*SADABS*; Bruker, 2009)	*h* = −15→15
*T*_min_ = 0.537, *T*_max_ = 0.953	*k* = −12→14
11390 measured reflections	*l* = −12→14

### Refinement

**Table:**

Refinement on *F*^2^	Secondary atom site location: difference Fourier map
Least-squares matrix: full	Hydrogen site location: inferred from neighbouring sites
*R*[*F*^2^ > 2σ(*F*^2^)] = 0.047	H atoms treated by a mixture of independent and constrained refinement
*wR*(*F*^2^) = 0.132	*w* = 1/[σ^2^(*F*_o_^2^) + (0.0702*P*)^2^ + 0.3162*P*] where *P* = (*F*_o_^2^ + 2*F*_c_^2^)/3
*S* = 1.05	(Δ/σ)_max_ < 0.001
3199 reflections	Δρ_max_ = 0.22 e Å^−^^3^
245 parameters	Δρ_min_ = −0.21 e Å^−^^3^
0 restraints	Extinction correction: *SHELXTL* (Sheldrick, 2008), Fc^*^=kFc[1+0.001xFc^2^λ^3^/sin(2θ)]^-1/4^
Primary atom site location: structure-invariant direct methods	Extinction coefficient: 0.0030 (5)

### Special details

**Table:**

Geometry. All e.s.d.'s (except the e.s.d. in the dihedral angle between two l.s. planes) are estimated using the full covariance matrix. The cell e.s.d.'s are taken into account individually in the estimation of e.s.d.'s in distances, angles and torsion angles; correlations between e.s.d.'s in cell parameters are only used when they are defined by crystal symmetry. An approximate (isotropic) treatment of cell e.s.d.'s is used for estimating e.s.d.'s involving l.s. planes.
Refinement. Refinement of *F*^2^ against ALL reflections. The weighted *R*-factor *wR* and goodness of fit *S* are based on *F*^2^, conventional *R*-factors *R* are based on *F*, with *F* set to zero for negative *F*^2^. The threshold expression of *F*^2^ > σ(*F*^2^) is used only for calculating *R*-factors(gt) *etc*. and is not relevant to the choice of reflections for refinement. *R*-factors based on *F*^2^ are statistically about twice as large as those based on *F*, and *R*- factors based on ALL data will be even larger.

### Fractional atomic coordinates and isotropic or equivalent isotropic displacement parameters (Å^2^)

**Table:**

	*x*	*y*	*z*	*U*_iso_*/*U*_eq_	
F1	1.08340 (11)	0.92571 (13)	0.19221 (15)	0.0946 (5)	
O1	0.59888 (10)	0.46944 (9)	0.14761 (10)	0.0507 (3)	
O2	0.85128 (13)	0.37355 (11)	−0.12356 (13)	0.0677 (4)	
N1	0.52279 (14)	0.57508 (14)	0.24812 (14)	0.0569 (4)	
N2	0.58458 (15)	0.85793 (13)	0.21110 (16)	0.0666 (4)	
C1	0.58225 (13)	0.57477 (13)	0.17674 (13)	0.0442 (3)	
C2	0.62297 (13)	0.66625 (13)	0.13969 (13)	0.0439 (3)	
C3	0.68193 (12)	0.66037 (12)	0.05215 (13)	0.0416 (3)	
H3A	0.6274	0.6888	−0.0262	0.050*	
C4	0.70666 (12)	0.53883 (12)	0.03481 (12)	0.0412 (3)	
C5	0.66493 (12)	0.45325 (12)	0.07952 (13)	0.0422 (3)	
C6	0.68132 (12)	0.33927 (13)	0.05802 (13)	0.0437 (3)	
C7	0.63629 (15)	0.24988 (14)	0.10278 (16)	0.0541 (4)	
H7A	0.5972	0.2650	0.1522	0.065*	
C8	0.64953 (19)	0.14210 (15)	0.07431 (19)	0.0656 (5)	
H8A	0.6191	0.0841	0.1040	0.079*	
C9	0.70845 (18)	0.11761 (15)	0.00090 (19)	0.0683 (5)	
H9A	0.7161	0.0435	−0.0187	0.082*	
C10	0.75491 (17)	0.20137 (15)	−0.04230 (17)	0.0592 (4)	
H10A	0.7947	0.1839	−0.0904	0.071*	
C11	0.74309 (13)	0.31434 (13)	−0.01470 (14)	0.0466 (4)	
C12	0.78985 (14)	0.40492 (14)	−0.05799 (14)	0.0490 (4)	
C13	0.77181 (13)	0.51312 (13)	−0.03431 (14)	0.0469 (4)	
H13A	0.8026	0.5710	−0.0638	0.056*	
C14	0.79036 (13)	0.73268 (12)	0.09176 (13)	0.0433 (3)	
C15	0.80585 (16)	0.80610 (15)	0.01145 (16)	0.0573 (4)	
H15A	0.7487	0.8125	−0.0664	0.069*	
C16	0.90543 (18)	0.87111 (17)	0.04453 (19)	0.0682 (5)	
H16A	0.9157	0.9203	−0.0102	0.082*	
C17	0.98691 (16)	0.86051 (16)	0.15903 (19)	0.0629 (5)	
C18	0.97531 (16)	0.78960 (18)	0.24158 (19)	0.0675 (5)	
H18A	1.0327	0.7844	0.3194	0.081*	
C19	0.87556 (15)	0.72490 (15)	0.20702 (16)	0.0567 (4)	
H19A	0.8663	0.6758	0.2624	0.068*	
C20	0.8921 (2)	0.46109 (19)	−0.1767 (2)	0.0726 (6)	
H20A	0.9294	0.4291	−0.2248	0.109*	
H20B	0.9463	0.5066	−0.1139	0.109*	
H20C	0.8282	0.5064	−0.2271	0.109*	
C21	0.60126 (14)	0.77220 (13)	0.17819 (14)	0.0479 (4)	
H2N1	0.4932 (18)	0.510 (2)	0.2566 (19)	0.065 (6)*	
H1N1	0.4912 (19)	0.642 (2)	0.251 (2)	0.073 (6)*	

### Atomic displacement parameters (Å^2^)

**Table:**

	*U*^11^	*U*^22^	*U*^33^	*U*^12^	*U*^13^	*U*^23^
F1	0.0660 (7)	0.0911 (9)	0.1290 (11)	−0.0335 (7)	0.0416 (7)	−0.0317 (8)
O1	0.0622 (7)	0.0378 (6)	0.0650 (7)	−0.0010 (5)	0.0391 (5)	−0.0020 (5)
O2	0.0845 (9)	0.0567 (8)	0.0838 (9)	0.0070 (6)	0.0566 (7)	−0.0061 (6)
N1	0.0703 (9)	0.0447 (8)	0.0724 (9)	−0.0022 (7)	0.0461 (8)	−0.0035 (7)
N2	0.0771 (10)	0.0491 (9)	0.0787 (10)	0.0032 (7)	0.0364 (8)	−0.0149 (7)
C1	0.0464 (7)	0.0404 (8)	0.0472 (8)	0.0021 (6)	0.0202 (6)	−0.0029 (6)
C2	0.0450 (7)	0.0399 (8)	0.0486 (8)	0.0018 (6)	0.0206 (6)	−0.0025 (6)
C3	0.0441 (7)	0.0378 (7)	0.0420 (7)	0.0014 (6)	0.0162 (6)	0.0007 (6)
C4	0.0429 (7)	0.0383 (7)	0.0411 (7)	0.0010 (5)	0.0155 (6)	−0.0019 (6)
C5	0.0444 (7)	0.0390 (7)	0.0440 (7)	0.0017 (6)	0.0185 (6)	−0.0025 (6)
C6	0.0433 (7)	0.0392 (8)	0.0443 (7)	0.0030 (6)	0.0131 (6)	−0.0014 (6)
C7	0.0605 (9)	0.0427 (8)	0.0615 (9)	0.0019 (7)	0.0269 (8)	0.0031 (7)
C8	0.0794 (12)	0.0396 (9)	0.0821 (12)	0.0006 (8)	0.0366 (10)	0.0040 (8)
C9	0.0804 (13)	0.0379 (9)	0.0857 (13)	0.0083 (8)	0.0322 (10)	−0.0052 (9)
C10	0.0653 (10)	0.0477 (9)	0.0664 (10)	0.0085 (8)	0.0283 (8)	−0.0084 (8)
C11	0.0460 (8)	0.0428 (8)	0.0471 (8)	0.0058 (6)	0.0146 (6)	−0.0042 (6)
C12	0.0494 (8)	0.0503 (9)	0.0499 (8)	0.0046 (7)	0.0227 (6)	−0.0053 (7)
C13	0.0506 (8)	0.0447 (8)	0.0488 (8)	−0.0014 (6)	0.0234 (6)	−0.0021 (6)
C14	0.0455 (7)	0.0367 (7)	0.0509 (8)	0.0004 (6)	0.0227 (6)	−0.0051 (6)
C15	0.0639 (10)	0.0539 (10)	0.0538 (9)	−0.0093 (8)	0.0232 (8)	0.0002 (7)
C16	0.0781 (13)	0.0595 (11)	0.0786 (12)	−0.0178 (9)	0.0434 (11)	−0.0029 (9)
C17	0.0534 (9)	0.0539 (10)	0.0883 (13)	−0.0127 (8)	0.0357 (9)	−0.0207 (9)
C18	0.0507 (10)	0.0709 (12)	0.0684 (11)	−0.0004 (8)	0.0107 (8)	−0.0124 (9)
C19	0.0566 (9)	0.0543 (10)	0.0550 (9)	−0.0006 (7)	0.0180 (7)	0.0028 (7)
C20	0.0858 (14)	0.0711 (13)	0.0848 (13)	−0.0002 (10)	0.0591 (12)	−0.0052 (10)
C21	0.0510 (8)	0.0442 (9)	0.0516 (8)	−0.0004 (6)	0.0237 (7)	−0.0039 (7)

### Geometric parameters (Å, º)

**Table:**

F1—C17	1.364 (2)	C8—C9	1.396 (3)
O1—C1	1.3440 (18)	C8—H8A	0.9300
O1—C5	1.3979 (18)	C9—C10	1.364 (3)
O2—C12	1.3627 (19)	C9—H9A	0.9300
O2—C20	1.425 (2)	C10—C11	1.411 (2)
N1—C1	1.349 (2)	C10—H10A	0.9300
N1—H2N1	0.89 (2)	C11—C12	1.427 (2)
N1—H1N1	0.90 (2)	C12—C13	1.361 (2)
N2—C21	1.147 (2)	C13—H13A	0.9300
C1—C2	1.356 (2)	C14—C15	1.376 (2)
C2—C21	1.411 (2)	C14—C19	1.378 (2)
C2—C3	1.517 (2)	C15—C16	1.394 (3)
C3—C4	1.516 (2)	C15—H15A	0.9300
C3—C14	1.525 (2)	C16—C17	1.357 (3)
C3—H3A	0.9800	C16—H16A	0.9300
C4—C5	1.356 (2)	C17—C18	1.358 (3)
C4—C13	1.419 (2)	C18—C19	1.392 (3)
C5—C6	1.415 (2)	C18—H18A	0.9300
C6—C7	1.413 (2)	C19—H19A	0.9300
C6—C11	1.418 (2)	C20—H20A	0.9600
C7—C8	1.359 (3)	C20—H20B	0.9600
C7—H7A	0.9300	C20—H20C	0.9600
			
C1—O1—C5	118.35 (12)	C9—C10—H10A	119.7
C12—O2—C20	116.83 (14)	C11—C10—H10A	119.7
C1—N1—H2N1	115.9 (14)	C10—C11—C6	118.83 (15)
C1—N1—H1N1	113.3 (15)	C10—C11—C12	122.65 (15)
H2N1—N1—H1N1	124.2 (19)	C6—C11—C12	118.51 (14)
O1—C1—N1	110.73 (14)	C13—C12—O2	124.33 (15)
O1—C1—C2	123.18 (13)	C13—C12—C11	120.91 (14)
N1—C1—C2	126.07 (15)	O2—C12—C11	114.76 (14)
C1—C2—C21	117.63 (13)	C12—C13—C4	120.87 (15)
C1—C2—C3	123.24 (13)	C12—C13—H13A	119.6
C21—C2—C3	118.96 (13)	C4—C13—H13A	119.6
C4—C3—C2	109.01 (12)	C15—C14—C19	118.55 (15)
C4—C3—C14	112.07 (12)	C15—C14—C3	120.19 (14)
C2—C3—C14	112.70 (12)	C19—C14—C3	121.25 (14)
C4—C3—H3A	107.6	C14—C15—C16	121.29 (17)
C2—C3—H3A	107.6	C14—C15—H15A	119.4
C14—C3—H3A	107.6	C16—C15—H15A	119.4
C5—C4—C13	118.64 (14)	C17—C16—C15	118.01 (18)
C5—C4—C3	122.12 (13)	C17—C16—H16A	121.0
C13—C4—C3	119.19 (13)	C15—C16—H16A	121.0
C4—C5—O1	123.18 (13)	C16—C17—C18	122.84 (17)
C4—C5—C6	122.86 (14)	C16—C17—F1	118.04 (19)
O1—C5—C6	113.93 (13)	C18—C17—F1	119.11 (19)
C7—C6—C5	123.01 (14)	C17—C18—C19	118.47 (17)
C7—C6—C11	118.83 (14)	C17—C18—H18A	120.8
C5—C6—C11	118.13 (14)	C19—C18—H18A	120.8
C8—C7—C6	120.61 (17)	C14—C19—C18	120.84 (17)
C8—C7—H7A	119.7	C14—C19—H19A	119.6
C6—C7—H7A	119.7	C18—C19—H19A	119.6
C7—C8—C9	120.59 (18)	O2—C20—H20A	109.5
C7—C8—H8A	119.7	O2—C20—H20B	109.5
C9—C8—H8A	119.7	H20A—C20—H20B	109.5
C10—C9—C8	120.56 (16)	O2—C20—H20C	109.5
C10—C9—H9A	119.7	H20A—C20—H20C	109.5
C8—C9—H9A	119.7	H20B—C20—H20C	109.5
C9—C10—C11	120.55 (17)	N2—C21—C2	179.06 (19)
			
C5—O1—C1—N1	−176.40 (13)	C9—C10—C11—C12	−179.82 (17)
C5—O1—C1—C2	2.4 (2)	C7—C6—C11—C10	1.6 (2)
O1—C1—C2—C21	−178.96 (14)	C5—C6—C11—C10	−176.68 (14)
N1—C1—C2—C21	−0.3 (2)	C7—C6—C11—C12	−179.03 (14)
O1—C1—C2—C3	5.8 (2)	C5—C6—C11—C12	2.7 (2)
N1—C1—C2—C3	−175.55 (15)	C20—O2—C12—C13	−4.3 (3)
C1—C2—C3—C4	−10.64 (19)	C20—O2—C12—C11	175.28 (16)
C21—C2—C3—C4	174.21 (13)	C10—C11—C12—C13	176.76 (16)
C1—C2—C3—C14	−135.72 (15)	C6—C11—C12—C13	−2.6 (2)
C21—C2—C3—C14	49.13 (18)	C10—C11—C12—O2	−2.8 (2)
C2—C3—C4—C5	8.57 (19)	C6—C11—C12—O2	177.82 (14)
C14—C3—C4—C5	134.03 (14)	O2—C12—C13—C4	179.97 (14)
C2—C3—C4—C13	−173.90 (12)	C11—C12—C13—C4	0.4 (2)
C14—C3—C4—C13	−48.44 (17)	C5—C4—C13—C12	1.6 (2)
C13—C4—C5—O1	−179.35 (13)	C3—C4—C13—C12	−176.02 (14)
C3—C4—C5—O1	−1.8 (2)	C4—C3—C14—C15	106.56 (16)
C13—C4—C5—C6	−1.4 (2)	C2—C3—C14—C15	−130.03 (15)
C3—C4—C5—C6	176.10 (13)	C4—C3—C14—C19	−72.26 (18)
C1—O1—C5—C4	−4.4 (2)	C2—C3—C14—C19	51.14 (19)
C1—O1—C5—C6	177.49 (12)	C19—C14—C15—C16	0.4 (3)
C4—C5—C6—C7	−178.91 (15)	C3—C14—C15—C16	−178.43 (16)
O1—C5—C6—C7	−0.8 (2)	C14—C15—C16—C17	−0.4 (3)
C4—C5—C6—C11	−0.7 (2)	C15—C16—C17—C18	0.1 (3)
O1—C5—C6—C11	177.36 (12)	C15—C16—C17—F1	−178.75 (17)
C5—C6—C7—C8	176.61 (16)	C16—C17—C18—C19	0.1 (3)
C11—C6—C7—C8	−1.6 (2)	F1—C17—C18—C19	178.99 (16)
C6—C7—C8—C9	0.4 (3)	C15—C14—C19—C18	−0.2 (3)
C7—C8—C9—C10	0.8 (3)	C3—C14—C19—C18	178.68 (15)
C8—C9—C10—C11	−0.7 (3)	C17—C18—C19—C14	−0.1 (3)
C9—C10—C11—C6	−0.5 (3)		

### Hydrogen-bond geometry (Å, º)

**Table:**

*D*—H···*A*	*D*—H	H···*A*	*D*···*A*	*D*—H···*A*
N1—H2*N*1···N2^i^	0.89 (2)	2.17 (2)	3.054 (2)	175 (2)

Symmetry code: (i) −*x*+1, *y*−1/2, −*z*+1/2.
